# A case report on enormous haemangioma of liver

**DOI:** 10.1097/MS9.0000000000001360

**Published:** 2023-10-04

**Authors:** Pratik Baral, Ramesh Singh Bhandari, Sumita Pradhan, Narendra Maharjan, Prakash Mainali, Dipesh Regmi, Krishna Kandel

**Affiliations:** Tribhuvan University Teaching hospital, Maharajgunj, Nepal

**Keywords:** angioembolization, Case report, hepatic hemangioma, Non-anatomical liver resection

## Abstract

**Introduction:**

Hemangiomas are the most common benign liver tumour. These tumours arise from the proliferation of vascular endothelial cells and increase in size owing to dilation. If their diameter exceeds 5 cm, they are classified as giant hemangiomas, while those surpassing 15 cm are considered enormous hemangiomas.

**Case presentation:**

A 38-year-old female patient presented with complaints of abdominal fullness for 18 months. Contrast-enhanced computed tomography was performed and two hemangiomas were diagnosed; the , larger one was an enormous hemangioma of size 20 × 16 cm. Non-anatomical hepatic resection was performed to remove the hemangiomas. The patient recovered well, without any complications.

**Clinical discussion:**

Hepatic hemangiomas are common, but hemangiomas greater than 15 cm in size are rare. They usually require no treatment unless the patient is symptomatic. Hepatectomy and enucleation of hemangioma are the most common surgical procedure for such hemangioma.

**Conclusions:**

Rarely, large hepatic hemangioma can be the cause of abdominal fullness lasting for months. Often, surgical intervention is required.

## Introduction

Highlights

- Hemangiomas are the most common benign tumour of liver.
- Hemangiomas exceeding 15 cm in greatest diameter are considered enormous hemangioma.
- Rarely, large hepatic hemangioma can be the cause of abdominal fullness lasting for months.
- Hepatectomy and enucleation of hemangioma are the most common surgical procedure for such hemangioma.

Hemangiomas are the most prevalent liver tumours, accounting for ~20% of cases^1^. However, only a small number of them grow to a size exceeding 5 cm, and only those larger ones may cause vague abdominal symptoms^2^. These neoplasms consist of groups of blood-filled cavities lined by endothelial cells, and they are thought to be vascular malformations that enlarge through dilation instead of hypertrophy or hyperplasia^3^. Haemangiomas are frequently discovered incidentally during imaging tests for other unrelated pathologies, and they are part of the “Incidentaloma” tumour group^1^. The cause of these tumours is not fully understood, but hormonal factors, such as exposure to oestrogen and progesterone during pregnancy or hormone replacement therapy, may play a role in their enlargement^4^. However, not all haemangiomas have oestrogen receptors, and some have been known to grow without hormonal replacement, even in post-menopausal women^1,4^. Due to the unpredictable growth pattern and ambiguous symptoms of liver haemangiomas, there is significant interest in their epidemiology and diagnosis, despite being a commonly found benign tumour. This case report has been reported in line with the SCARE (Surgical Case Report) criteria^5^.

### Case presentation

A 38-year-old woman presented to our institution Out-Patient Department with complain of abdominal mass for last one and half year. She also reported experiencing abdominal fullness, shortness of breath, and loss of appetite in the past one to two months but denied experiencing any abdominal pain, jaundice, or irregular menstrual flow, had no family history of liver diseases, and was not taking any medications. Upon physical examination, a large, non-tender, firm mass measuring around 20 ×15 cm was detected in the right hypochondriac region extending towards the umbilical region.

**Figure 1:**
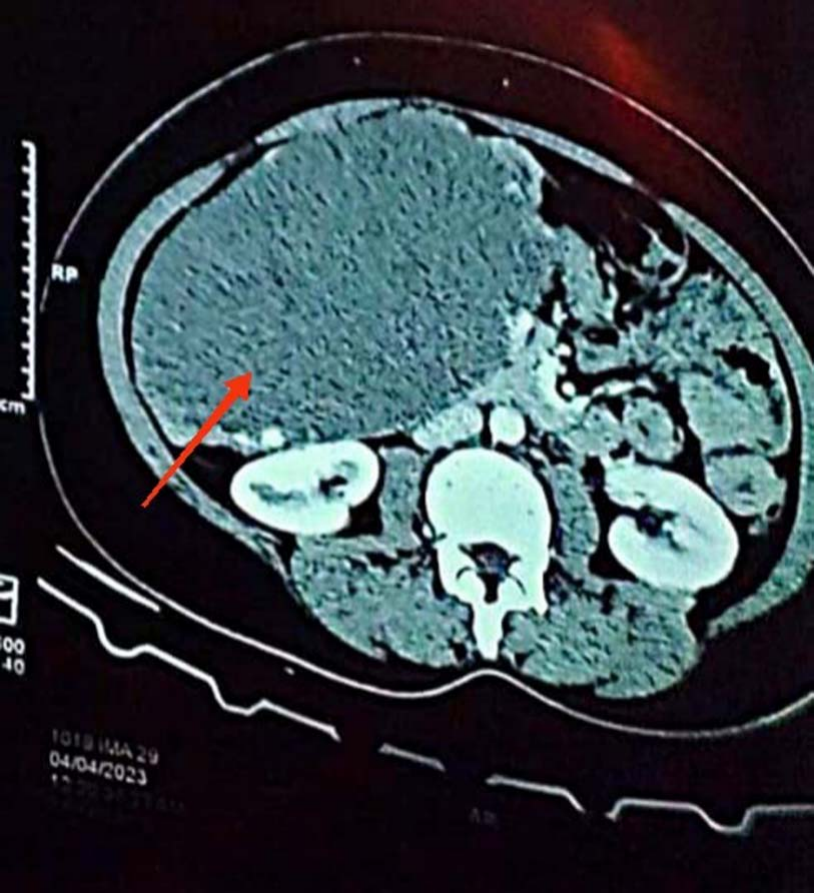
Computed tomography of abdomen showing hypodense lesion (hemangioma) in liver by red arrow.

Ultrasonography of abdomen was done which revealed an ill-defined, heterogeneous isoechoic lesion measuring ~13 ×12 cm with a central hypoechoic area and internal vascularity. Suspecting hepatic hemangioma, contrast -enhanced computed tomography was done which showed two well-defined hypodense lesions in the liver, one measuring 19 ×18 × 12 cm and exophytic in nature, arising from segments V, VI, and IVb(Figure 1). The larger lesion abutted the common bile duct and portal vein superiorly, compressed and displaced the gallbladder anteromedially, compressed the right kidney posteromedially, and abutted the anterior abdominal wall and displaced the pancreas and small bowel loops medially. The lesion also compressed the inferior vena cava posteriorly. The smaller lesion, measuring 4.7 ×4.4 ×4.3 cm, was located in segments II and III of the left lobe of the liver and exhibited similar enhancement characteristics suggestive of hepatic hemangiomas. Complete blood count, liver function tests, and coagulation profiles were normal, as were tumour markers, including carcinoembryonic antigen, CA-19-9 and alpha-fetoprotein.

### Treatment

**Figure 2:**
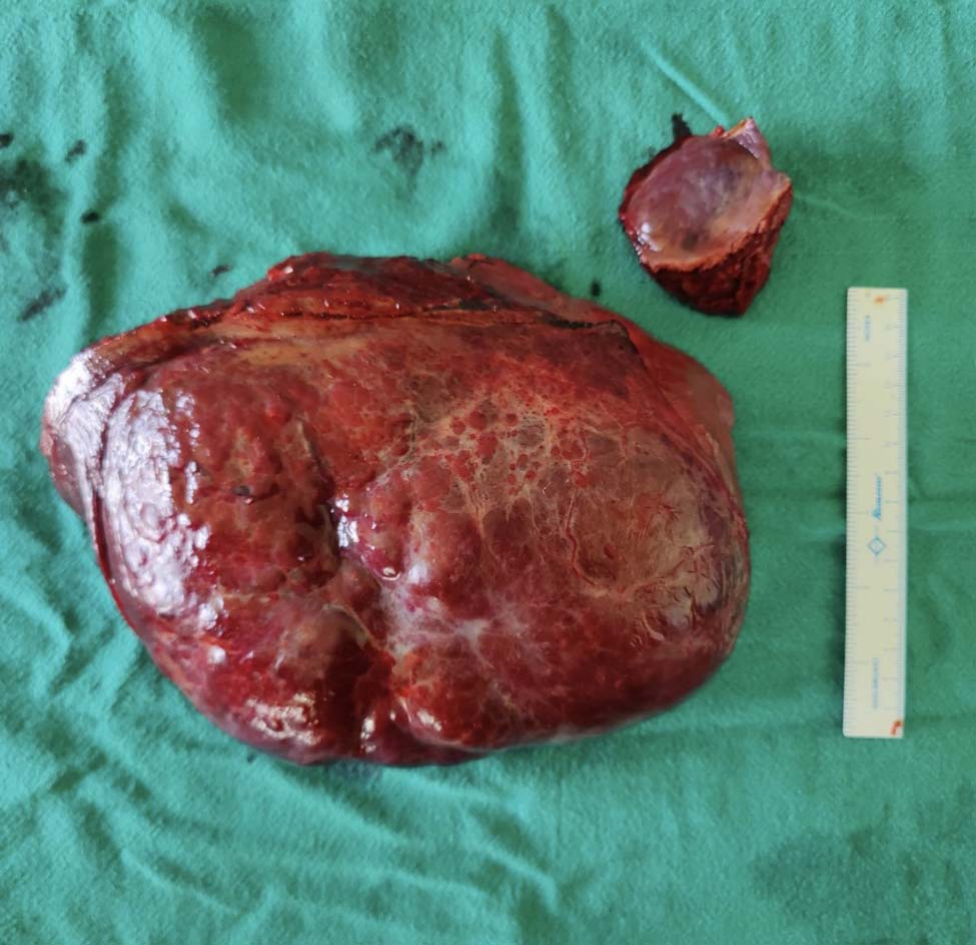
Hepatic hemangiomas (large one is enormous hemangioma, smaller one is giant hemangioma).

The patient underwent non-anatomical hepatic resection following angioembolisation performed the day before under ultrasonic fluoroscopic guidance. Angioembolisation was done to minimize the blood loss, especially in case it ruptures during surgery. Non-anatomical resection of liver was done using Kelly and clamp method and harmonic scalpel. The hemangiomas were measured 20×16 cm and 5×5 cm (Figure 2). The larger one being involved in segments IVB, V and VI . The smaller one involved in segment II and III. Gallbladder was also removed as hemangioma was extending to segment IVB.

### Outcome and follow-up

The patient recovered well from surgery. Postoperative follow-up to present has been uneventful.

## Discussion

Hepatic hemangiomas are the most common benign tumours of the liver^1^. They are mesenchymal in origin and are usually solitary^6^. They are usually discovered incidentally on imaging studies done for other purposes^1^. The most common location is the right lobe of the liver and are more common in women with a female to male ratio of 5:1^7^. This is due to high exposure to oestrogen and progesterone stimulates the growth of these tumour^4^. Most hepatic hemangiomas are small and asymptomatic, but in rare cases, they can grow to enormous sizes and cause abdominal discomfort and other complications^2^. Typical hemangiomas, known as capillary hemangiomas, vary in size from a few millimetres to 3 cm. They do not grow larger over time and are therefore unlikely to cause future symptoms. Small (mm–3 cm) and medium (3–10 cm) hemangiomas are well defined and do not require treatment apart from regular follow-up. However, giant liver hemangiomas, reaching upto 10 cm or even exceeding 15 cm, termed as enormous hemangioma, often develop symptoms and complications^1^.

Di Carlo and colleagues’ 2016 study defined four size categories of hepatic hemangioma. They reviewed reports from 1970 to 2014 and found 154 patients with haemangiomas smaller than 5 cm (small), 182 between 5 and 9.9 cm (large), 75 between 10 and 14.9 cm (giant), and 102 exceeding 15 cm (enormous). They concluded that the term ‘giant’ should be used for tumours larger than 10 cm, and surgery should only be considered if the patient experiences symptoms like abdominal discomfort or haematological alterations^8^.

In this case report, we presented the case of a 38-year-old woman who had an enormous hepatic hemangioma measuring 20×16 cm . Ribeiro and colleagues’ literature review suggests that in healthy patients without comorbidities, surgical resection is universally recommended for true giant liver hemangiomas larger than 10 cm. This is due to the risk of catastrophic rupture, especially when they are situated in areas prone to abdominal rupture. Surgery is absolutely necessary in cases of spontaneous or traumatic rupture with hemoperitoneum, intratumoral bleeding, and the extremely rare Kasabach–Merrit syndrome^9^. For centrally located giant hemangiomas, resection is preferred over enucleation to minimize the risk of significant bleeding. Percutaneous ablation is an option only for lesions smaller than 5 cm, and in such cases, observation is favored over intervention, resulting in the infrequent use of this approach^1^. In our case, we opted for a non-anatomical resection of the liver using the Kelly and clamp method and harmonic scalpel after angioembolization. This approach has been shown to be safe and effective for treating large hemangiomas and minimizing blood loss during surgery^10^.

## Conclusion

We presented a rare case of an enormous hepatic hemangioma in 38-year-old lady with symptom of abdominal fullness. Sometimes, it may exceeds 15 cm in size requiring surgical treatment. The successful management of this lesion using a non-anatomical resection after embolisation of feeding vessel highlights the importance of careful planning and coordination between interventional radiology and surgery teams.

## Ethical approval

Not required for a case report in our institution.

## Consent

Written informed consent was obtained from the patient for publication of this case report and accompanying images. A copy of the written consent is available for review by the Editor-in-Chief of this journal on request

## Sources of funding

The authors received no financial support for the research, authorship, and/or publication of this article.

## Author contribution

P.B.: wrote the original manuscript, reviewed, and edited the original manuscript. P.M., D.R. and K.K.: reviewed the original manuscript. S.P. and N.M.: reviewed and edited the original manuscript. R.S.B.: senior author and manuscript reviewer.

## Conflicts of interest disclosure

Authors have no conflict of interest to declare.

## Research registration unique identifying number (UIN)

Name of registry : None.

Unique Identifying number or registration ID : None.

Hyperlink to your specific registration (must be publicly accessible and will be checked): None.

## Guarantor

Pratik Baral Is guarantor for this manuscript.

## Data availability statement

All available data are within the manuscript itself.

## Provenance and peer review

Not commissioned, externally peer- reviewed.

## Acknowledgements

The authors express their gratitude to the patient for granting consent for the publication of this case report. Additionally, the authors extend their thanks to the Department of Gastrointestinal and Hepatobiliary Surgery of their institution, as well as to all the healthcare professionals, including doctors and nurses, who were involved in the patient’s care. Without their support and dedication, this report would not have been possible.
